# Simultaneous Transcatheter Aortic Valve Replacement and Endovascular Aortic Aneurysm Repair—The First Case in Serbia

**DOI:** 10.3390/diagnostics15212785

**Published:** 2025-11-03

**Authors:** Darko Boljević, Jovana Lakčević, Mihajlo Farkić, Vladimir Mihajlović, Stefan Veljković, Armin Šljivo, Marina Lukić, Milovan Bojić, Aleksandra Nikolić

**Affiliations:** 1 Cardiovascular Institute “Dedinje”, 11040 Belgrade, Serbiavlada_sd@yahoo.com (V.M.);; 2 Clinical Center of University of Sarajevo, 71000 Sarajevo, Bosnia and Herzegovina; 3 Faculty of Medicine, University of Banja Luka, 78000 Banja Luka, Bosnia and Herzegovina; 4 Faculty of Medicine, University of Belgrade, 11040 Belgrade, Serbia

**Keywords:** aortic stenosis, AAA, TAVR, EVAR, Serbia

## Abstract

**Background and Clinical Significance**: Concomitant severe aortic stenosis (AS) and abdominal aortic aneurysm (AAA) in elderly patients presents a significant therapeutic challenge. While transcatheter aortic valve replacement (TAVR) and endovascular aneurysm repair (EVAR) have become established minimally invasive treatments for high-risk patients, simultaneous management of both conditions remains rare. **Case Presentation**: We report the first documented case in Serbia of a simultaneous TAVR and EVAR in a 75-year-old male with severe symptomatic AS and AAA. The patient had a history of hypertension, diabetes mellitus, atrial fibrillation, prior radiofrequency pulmonary vein ablation, and pacemaker implantation. Echocardiography demonstrated severe AS with a transvalvular gradient of 116/61 mmHg, an aortic valve area of 0.6 cm^2^, and a left ventricular ejection fraction of 30–35%. Coronary angiography revealed 50–60% stenosis of the right coronary artery. Following evaluation by a multidisciplinary Heart and Vascular Team, a combined procedure was performed under general anesthesia via bilateral femoral access. TAVR with a Medtronic Evolut R valve was successfully deployed, followed by EVAR with satisfactory stent graft positioning and angiographic results. The patient’s postoperative course was uneventful, and he was discharged on the ninth day. At six-month follow-up, echocardiography showed optimal valve function, and CT identified a type II endoleak, which was managed conservatively. **Conclusions**: This case demonstrates the feasibility and safety of simultaneous TAVR and EVAR in a high-risk elderly patient, emphasizing the importance of careful preoperative planning and a coordinated multidisciplinary approach. Further studies are warranted to establish standardized guidelines for the management of patients with coexisting severe AS and AAA.

## 1. Introduction

Aortic stenosis (AS) represents the most prevalent valvular heart disease in developed countries, with a reported prevalence of approximately 12.4% among individuals aged 75 years and older, while severe AS affects around 3.4% in this age group [1]. For severe, symptomatic AS, aortic valve replacement remains the only definitive therapy, either by surgical aortic valve replacement (SAVR) or transcatheter aortic valve replacement (TAVR) [2]. Since its introduction in 2002, TAVR has marked a paradigm shift in the treatment of AS, evolving from a therapy reserved for inoperable or high-surgical-risk patients to a guideline-endorsed option across all surgical risk categories, supported by robust evidence from randomized controlled trials and large real-world registries [3].

Similarly, the management of abdominal aortic aneurysms (AAAs) has undergone a transformative change. Since 2003, endovascular aortic aneurysm repair (EVAR) has surpassed open surgical repair as the predominant treatment strategy for infrarenal AAAs, largely because it results in reduced perioperative morbidity and mortality, shorter hospital stay, and faster recovery [4].

Although epidemiological data on the coexistence of severe AS and AAA remain limited, available reports suggest that the prevalence of concomitant disease in elderly populations may exceed 6% [4,5]. This association is not unexpected given that both conditions share common risk factors, including advanced age, hypertension, dyslipidemia, and systemic atherosclerosis. The coexistence of these two pathologies presents a unique clinical challenge, as both require timely intervention to prevent catastrophic outcomes such as rupture in the case of AAA and sudden cardiac death in the case of untreated severe AS.

From a technical perspective, both TAVR and EVAR most commonly employ transfemoral arterial access [6,7]. This anatomical and procedural overlap theoretically facilitates a combined approach; however, simultaneous performance of TAVR and EVAR remains exceedingly rare. To date, only a small number of high-volume cardiovascular centers worldwide have reported successful cases of concomitant TAVR and EVAR, reflecting both the technical complexity of the strategy and the need for multidisciplinary collaboration between structural heart disease specialists, vascular surgeons, and anesthesiologists [8,9]. Emerging case reports and series highlight the potential feasibility and safety of this strategy, yet robust evidence regarding long-term outcomes is lacking [9]. Further multicenter studies and registry data are needed to better define patient selection, procedural sequencing, and perioperative management in this high-risk population.

The aim of this case report is to present the first documented instance in Serbia of a simultaneous TAVR and EVAR, describing the clinical presentation, procedural strategy, and short-term outcomes in an elderly patient.

## 2. Case Presentation

A 75-year-old male was referred to our institution for evaluation and management of severe AS. His medical history was significant for hypertension, diabetes mellitus, atrial fibrillation, and benign prostatic hyperplasia. He had previously undergone radiofrequency ablation of the pulmonary veins in 2009 and implantation of a permanent pacemaker in 2017. The patient reported progressive exertional dyspnea and fatigue. Prior to diagnostic testing, the patient was receiving the following pharmacological therapy: acenocoumarol 0.25 tablet once daily, bisoprolol 50 mg twice daily, flecainide 100 mg three times daily, irbesartan/hydrochlorothiazide 20/25 mg once daily, lisinopril/amlodipine 20/10 mg once daily, atorvastatin 20 mg once daily, betamethasone 0.4 mg once daily, alprostadil 5 mg once daily, and metformin 1000 mg once daily. The complete blood count showed the following values: leukocytes 6.5 × 10^9^/L, neutrophils 64.4%, lymphocytes 20.0%, monocytes 9.1%, eosinophils 5.3%, and basophils 0.4%. The absolute neutrophil count was 4.2 × 10^9^/L, lymphocytes 1.3 × 10^9^/L, monocytes 0.6 × 10^9^/L, eosinophils 0.3 × 10^9^/L, and basophils 0.0 × 10^9^/L. The red blood cell count was 3.96 × 10^12^/L, hemoglobin 117 g/L, hematocrit 0.361 L/L, mean corpuscular volume 91.2 fL, mean corpuscular hemoglobin 29.6 pg, and mean corpuscular hemoglobin concentration 324 g/L. Red cell distribution width was 15.7%. Platelet count was 192 × 10^9^/L, mean platelet volume 10.4 fL, plateletcrit 0.200%, and platelet distribution width 17.7%.

The biochemical profile revealed the following results: sodium 141 mmol/L, potassium 4.1 mmol/L, calcium 2.23 mmol/L, magnesium 0.71 mmol/L, chloride 102 mmol/L, phosphorus 0.99 mmol/L, and total carbon dioxide 27.67 mmol/L. Total bilirubin was 10.4 µmol/L, urea 9.10 mmol/L, creatinine 102.2 µmol/L, and glucose 6.92 mmol/L. Alanine aminotransferase was 15 IU/L, aspartate aminotransferase 13 IU/L, lactate dehydrogenase 202 IU/L, creatine kinase 49 IU/L, and CK-MB 14 IU/L. Total protein was 86.93 g/L, albumin 36.0 g/L, and high-sensitivity troponin I 10.3 pg/mL.

**Figure 1:**
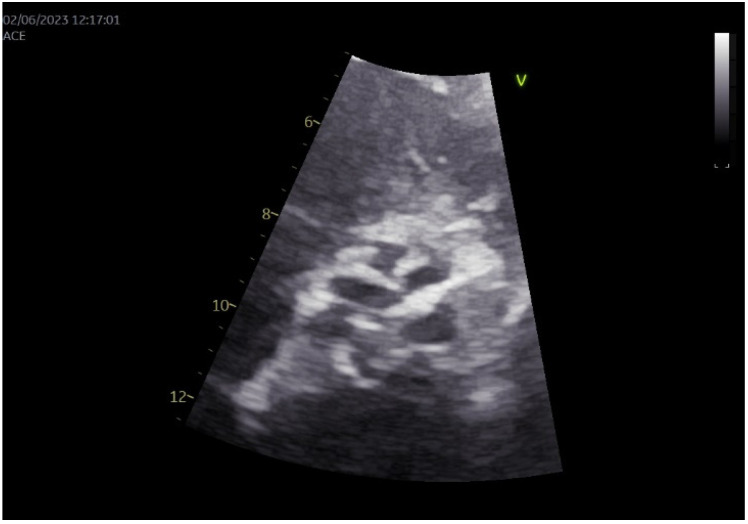
A focused transthoracic echocardiographic examination using GE^®^ Vivid E95 (GE HealthCare, Chicago, IL, USA) in the short-axis view demonstrated a heavily calcified aortic valve.

Transthoracic echocardiography demonstrated severe AS, with a transvalvular pressure gradient of 116/61 mmHg, an aortic valve area of 0.6 cm^2^ (indexed to 0.23 cm^2^/m^2^), and globally reduced left ventricular ejection fraction (30–35%) (Figure 1). Coronary angiography revealed 50–60% stenosis of the right coronary artery.

**Figure 2:**
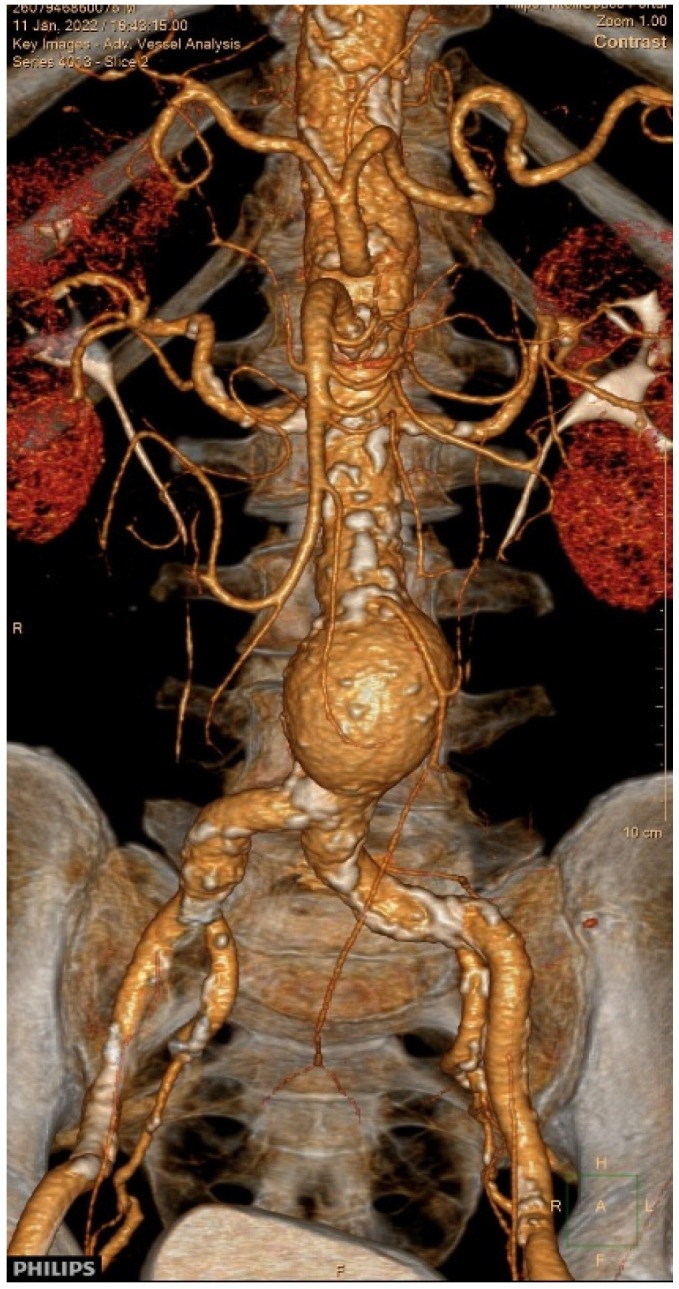
Three-dimensional computed tomography reconstruction of the abdominal aortic aneurysm.

As part of the pre-procedural evaluation for TAVR, computed tomography (CT) of the aorta was performed, which incidentally identified an AAA measuring 46 mm in diameter (Figure 2).

Following multidisciplinary evaluation, the Heart Team recommended TAVR due to the presence of a porcelain aorta, while the Vascular Team indicated EVAR for the AAA. After careful consideration, both teams concluded that a simultaneous TAVR and EVAR procedure would be the most appropriate approach for this high-risk patient.

**Figure 3:**
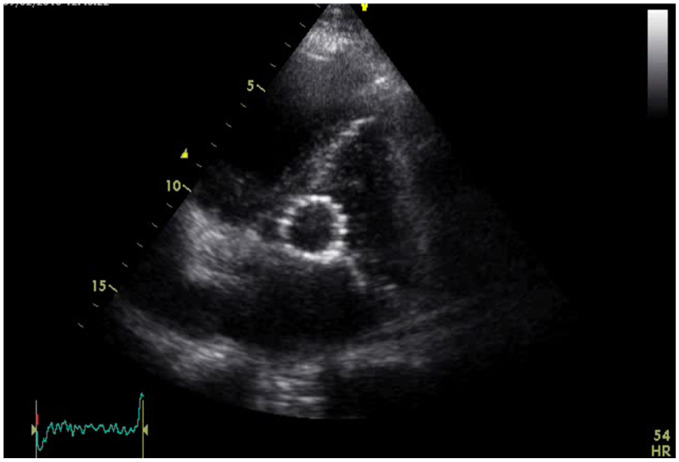
Medtronic^®^ Evolut R transcatheter aortic valve in its final aortic position.

The patient underwent the combined procedure under general anesthesia. Both femoral arteries were surgically exposed to facilitate vascular access. A guiding wire was introduced via the right femoral artery, while a 20 French sheath was inserted into the left femoral artery. A Safari Small wire was advanced to the left ventricular apex to enable rapid ventricular pacing. Balloon predilatation was performed using a 28 × 40 mm balloon, followed by deployment of a self-expanding Evolut R valve No. 34 (Medtronic^®^) in the aortic position (Figure 3).

Subsequently, EVAR was performed, achieving optimal prosthesis positioning and satisfactory angiographic results. Hemostasis was achieved by direct suture closure of both femoral arteries. At hospital discharge, the patient was prescribed the following therapy: acenocoumarol 4 mg adjusted according to the INR target range between 2 and 3; bisoprolol 5 mg, half a tablet twice daily; spironolactone 100 mg, half a tablet daily (In combination with furosemide as Edemid forte); sacubitril/valsartan 51 mg/49 mg, two tablets twice daily; empagliflozin 10 mg once daily; furosemide 10 mg once daily; ranolazine 10 mg, two tablets daily (at 13:00 and 18:00); and rosuvastatin 20 mg once daily.

**Figure 4:**
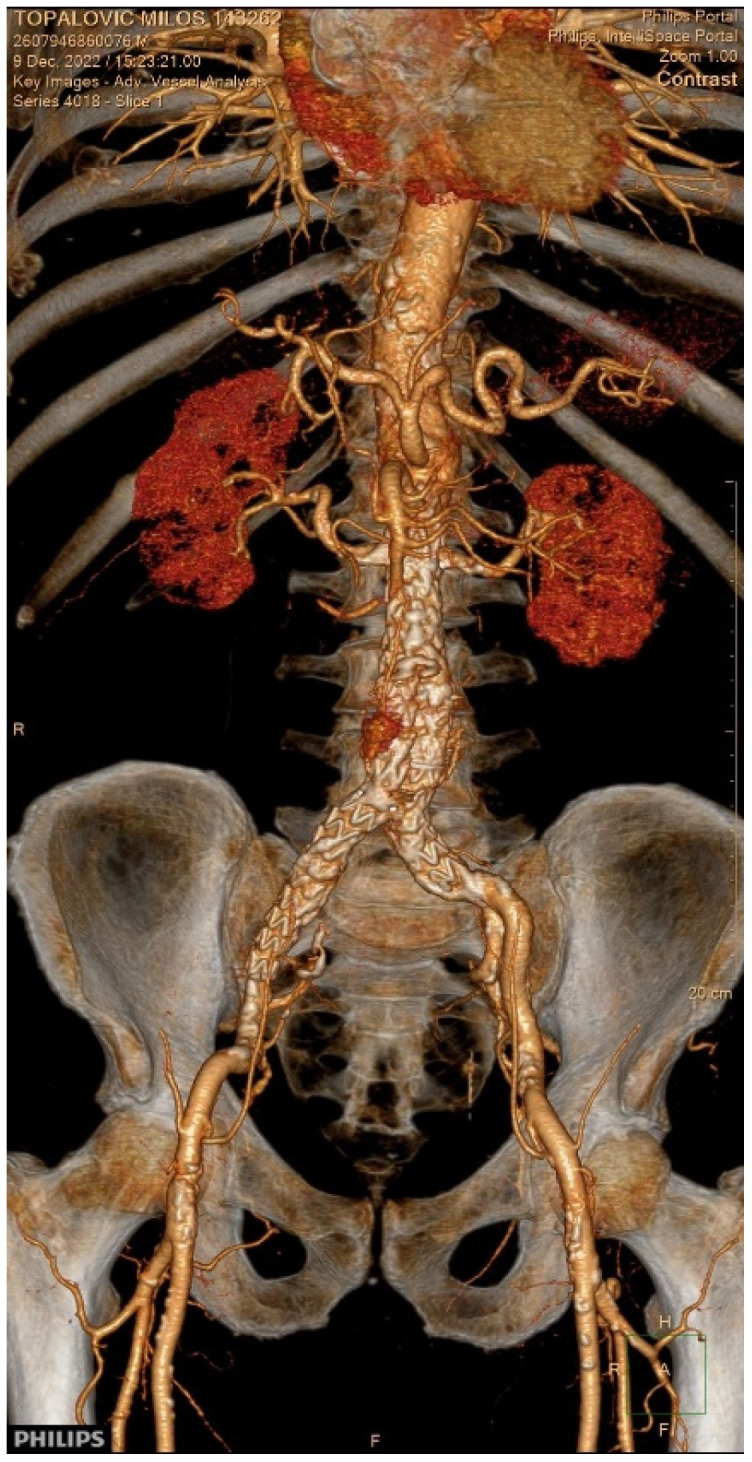
Three-dimensional computed tomography reconstruction of EVAR demonstrating a type II endoleak from the lumbar artery.

At the scheduled outpatient follow-up, the patient demonstrated clinical improvement. Echocardiographic assessment revealed a transvalvular pressure gradient of 22/13 mmHg across the prosthetic aortic valve, with only trace aortic regurgitation. Six-month follow-up CT of the aorta identified a type II endoleak originating from the lumbar artery (Figure 4). After multidisciplinary evaluation, the Vascular Team recommended a conservative management approach. Endoleak was treated conservatively with regular clinical and imaging follow-ups to monitor for changes or progression.

## 3. Literature Review and Discussion

### 3.1. Search Strategy

We conducted a systematic literature search to identify all available reports describing simultaneous TAVR and EVAR in adult patients. A comprehensive search of the literature was conducted for publications from 2010 through 1 October 2025 using the terms “TAVR,” “EVAR,” “simultaneous,” and “combined procedure”. To capture a wider range of relevant studies, additional synonymous and related terms were incorporated, and the reference lists of pertinent reviews and eligible articles were manually screened for further citations [10,11,12,13,14,15,16,17,18,19,20,21,22,23,24,25,26,27,28,29].

### 3.2. Definition, Epidemiology, and Demographics

Simultaneous TAVR and EVAR presents percutaneous treatment of AS and AAA during the same procedural session, which has been increasingly reported in elderly, high-risk patients with multiple comorbidities for whom conventional surgery carries excessive risk. A recent multicenter European study found that among 44 patients needing both procedures, 57% underwent them simultaneously, highlighting the increasing feasibility of this combined approach [10]. An analysis of the U.S. National Inpatient Sample from 2018 to 2021 reported 270 patients treated with simultaneous TAVR–EVAR and 70 with staged procedures, finding that simultaneous treatment was linked to shorter hospital stays without an increase in in-hospital mortality [11].

### 3.3. Justification for Combined Interventions, and Strategic Considerations in Procedural Sequencing

Because the complex hemodynamic interplay between AS and AAA makes management challenging, combined TAVR–EVAR is considered when both lesions are symptomatic or meet intervention criteria, aiming to minimize repeated vascular access, multiple admissions, and anesthetic exposure, and also to reduce hospital stays and costs. In severe AS, reduced cardiac output may protect aneurysms from rupture; however, following TAVR, the sudden drop in afterload and rise in systolic pressure could precipitate it [9]. Conversely, performing EVAR first may induce hypotension from aortic clamping or contrast, potentially worsening myocardial perfusion in severe AS and triggering ischemia [12,15,16].

#### 3.3.1. TAVR-First Strategy

Performing TAVR before EVAR offers several advantages, including rapid relief of outflow obstruction that improves cardiac output prior to EVAR, a lower risk of intraoperative hypotension during EVAR, better tolerance of anesthesia, and the ability to use the same femoral access for the subsequent procedure. However, this sequence also carries potential drawbacks, such as post-TAVR hypertension increasing aortic wall stress and the risk of aneurysm rupture, heightened pulsatility that may dislodge mural thrombus and cause embolization, and the possibility of injury to aneurysmal ilio-femoral arteries from large TAVR sheaths [9,17,18].

#### 3.3.2. EVAR-First Strategy

Contrarily, performing EVAR before TAVR provides distinct procedural benefits such as stabilizing the aneurysm to prevent rupture during or after TAVR, avoiding exposure to high arterial pressure during TAVR, and reducing the potential embolic risk from aneurysm thrombus. However, this approach also has disadvantages. Because systemic hypotension and contrast load may compromise coronary perfusion and trigger hemodynamic collapse in severe AS, the recovery period may delay TAVR and leave the patient symptomatic, and additional vascular access is required. Although cases of staged EVAR followed by TAVR have been reported, careful hemodynamic monitoring and collaborative planning are essential [12,19].

### 3.4. Procedural Sequencing and Complications

In a TAVR-first strategy, the valve is delivered through transfemoral access, and once its position and hemodynamic stability are confirmed, the sheath is exchanged for the EVAR device, with contrast use minimized and heparin reversed only after completing EVAR. In an EVAR-first approach, the aneurysm is repaired first, followed by TAVR either immediately after achieving hemostasis or on a separate day as a staged procedure. Performing both procedures simultaneously can reduce overall contrast exposure by allowing a single injection for both devices, though careful attention is required to prevent contrast-induced nephropathy.

Acute kidney injury (AKI), predominantly associated with contrast-induced nephrotoxicity and peri-procedural hypotension, represents a major procedural complication that can be mitigated through adequate intravenous hydration, avoidance of nephrotoxic medications, limitation of contrast load, utilization of iso-osmolar contrast agents, and implementation of low-contrast imaging protocols or adjunctive intravascular ultrasound guidance [13,20].

Severe calcification and mural thrombus in the aneurysm or aortic arch increase the risk of embolization during wire and device manipulation, but this risk can be reduced by using cerebral protection devices, ensuring proper anticoagulation, and carefully flushing the catheters.

### 3.5. Post-Procedural Care

Patients should be monitored in an intensive care or step-down unit for hemodynamic stability, arrhythmias, bleeding, and renal function. Early ambulation and discharge (within 5–7 days) are feasible; the Italian study reported shorter hospital stays after simultaneous procedures compared with staged ones [10,21]. Secondary prevention includes antiplatelet therapy (aspirin ± clopidogrel), statins, blood pressure control, and smoking cessation. Lifelong surveillance imaging is mandatory for EVAR to detect endoleaks, graft migration, and aneurysm sac enlargement; CT angiography at 1 month, 6 months and annually is typical.

### 3.6. Prognosis and Follow-Up

#### 3.6.1. Short-Term Outcomes

Observational data suggest that simultaneous TAVR–EVAR has acceptable safety compared with staged interventions. The Italian multicenter study reported no difference in 30-day mortality between simultaneous and staged procedures (0% vs. 5%) [10]. Pulmonary complications (e.g., pneumonia) and permanent pacemaker implantation were less frequent in the simultaneous group [10]. The National Inpatient Sample analysis showed similar in-hospital mortality but shorter lengths of stay, lower costs, fewer cases of AKI, and fewer vascular complications with simultaneous procedures [11]. In a small staged EVAR-then-TAVR series (*n* = 5), no 30-day deaths or major complications occurred. Case reports of simultaneous TAVR–EVAR under regional anesthesia described stable hemodynamics and prompt recovery [12].

#### 3.6.2. Long-Term Outcomes

Long-term data are limited. The Italian registry reported a 3-year survival of 73% with no significant difference between simultaneous and staged groups [10]. Rehospitalization for heart failure or vascular complications occurred in 27% of patients. Pacemaker implantation was an independent predictor of mortality [10]. In the staged EVAR-then-TAVR case series, all patients remained alive at median follow-up of 21 months without aneurysm rupture or endoleak. Data from the general TAVR population indicate excellent valve durability, with structural valve deterioration < 3% at 5 years; whether concomitant EVAR influences durability remains unknown [22,23,24].

### 3.7. Surveillance

Post-EVAR surveillance aims to detect endoleaks, graft migration, and aneurysm sac enlargement [25]. CTA or contrast-enhanced ultrasound is recommended at 1 month, 6 months, and annually thereafter; some centers extend intervals after 2 years if the sac is stable. Post-TAVR follow-up [26] involves echocardiographic assessment at discharge, after 30 days, at 6 months, and annually thereafter, with particular attention to paravalvular regurgitation, transvalvular gradients, and ventricular performance. Long-term secondary prevention relies on lifelong antiplatelet therapy, statin administration, strict blood-pressure and lipid control, and smoking cessation.

### 3.8. Our Case

This case adds to the limited but growing body of evidence supporting the feasibility of combined TAVR–EVAR interventions in high-risk patients. This body of evidence dates back to the first report of sequential procedures in 2012 [14]. The coexistence of severe AS and AAA is increasingly recognized in elderly men with an extensive atherosclerotic burden, reflecting overlapping pathophysiological mechanisms [26,27,28,29].

## 4. Conclusions

This first documented simultaneous TAVR and EVAR in Serbia demonstrates that a combined approach is feasible and safe in high-risk elderly patients with severe AS and AAA. Success relies on meticulous preoperative planning and multidisciplinary coordination. While short-term outcomes were favorable, long-term follow-up is essential, and further studies are needed to guide standardized management of these complex cases.

## Data Availability

Data are available upon request.

## References

[1] Osnabrugge R.L., Mylotte D., Head S.J., Van Mieghem N.M., Nkomo V.T., LeReun C.M., Bogers A.J., Piazza N., Kappetein A.P. (2013). Aortic stenosis in the elderly: Disease prevalence and number of candidates for transcatheter aortic valve replacement: A meta-analysis and modeling study. J. Am. Coll. Cardiol..

[2] Mahmaljy H., Tawney A., Young M. (2023). Transcatheter Aortic Valve Replacement.

[3] Jaegere P., Ronde M., Heijer P., Weger A., Baan J. (2020). The history of transcatheter aortic valve implantation: The role and contribution of an early beliver and adopter, the Netherlands. Neth. Heart J..

[4] Mauri S., Bozzani A., Ferlini M., Aiello M., Gazzoli F., Pirrelli S., Valsecchi F., Ferrario M. (2019). Combined Transcatheter Treatment of Severe Aortic Valve Stenosis and Infrarenal Abdominal Aortic Aneurysm in Increased Surgical Risk Patients. Ann. Vasc. Surg..

[5] Koutsias S., Karaolanis G.I., Papafaklis M.I., Peroulis M., Tzimas P., Lakkas L., Mitsis M., Naka K.K., Michalis L.K. (2020). Simultaneous Transcatheter Aortic Valve Implantation and Infrarenal Aortic Aneurysm Repair for Severe Aortic Stenosis and Abdominal Aortic Aneurysm: Report of 2 Cases and Literature Review. Vasc. Endovasc. Surg..

[6] Harloff M.T., Percy E.D., Hirji S.A., Yazdchi F., Shim H., Chowdhury M., Malarczyk A.A., Sobieszczyk P.S., Sabe A.A., Shah P.B. (2020). A step-by-step guide to trans-axillary transcatheter aortic valve replacement. Ann. Cardiothorac. Surg..

[7] England A., Williams R.M. (2013). Endovascular Aortic Aneursym Repair (EVAR). Ulster Med. J..

[8] Schizas N., Antonopoulous C.N., Patris V., Lampropoulous K., Kratimenos T., Argiriou M. (2021). Current issues on simultaneous TAVR (Transcatheter Aortic Valve Replacement) and EVAR (Endovascular Aneurysm Repair). Clin. Case Rep..

[9] Medda M., Casilli F., Bande M., Glauber M., Tespili M., Cirri S. (2023). Percutaneous treatment of abdominal aortic aneurysm and aortic valve stenosis with ‘staged’ EVAR and TAVR: A case series. J. Cardiothorac. Surg..

[10] Gallitto E., Spath P., Faggioli G.L., Saia F., Palmerini T., Piazza M., D’Oria M., Simonte G., Cappiello A., Isernia G. (2024). Simultaneous versus staged approach in transcatheter aortic valve implantation for severe stenosis and endovascular aortic repair for thoracic and abdominal aortic aneurysm. Eur. J. Cardiothorac. Surg..

[11] Pu A., Annabathula R.V., Allaham H., Chahal D., Toursavadkohi S., Gupta A. (2025). In-Hospital Outcomes of Simultaneous and Staged Transcatheter Aortic Valve Replacement and Endovascular Aneurysm Repair. Catheter. Cardiovasc. Interv..

[12] Zaccarelli M., Testa T.S., Buscaglia G., Pratesi G., Crimi G., Balbi M., Di Gregorio S., Silvetti S. (2024). Anesthetic Considerations in Combined TAVR and Aortic Endovascular Procedures, a Case Report. Ann. Card. Anaesth..

[13] Jhaveri K.D., Saratzis A.N., Wanchoo R., Sarafidis P.A. (2017). Endovascular aneurysm repair (EVAR)—A transcatheter aortic valve replacement (TAVR)—Associated kidney injury. Kidney Int..

[14] Drury-Smith M., Garnham A., Khogali S. (2012). Sequential trans-catheter aortic valve implantation and abdominal aortic aneurysm repair. Catheter. Cardiovasc. Interv..

[15] Drury-Smith M., Garnham A., Khogali S. (2012). Critical aortic stenosis in a patient with a large saccular abdominal aortic aneurysm: Simultaneous transcatheter aortic valve implantation and drive-by endovascular aortic aneurysm repair. Catheter. Cardiovasc. Interv..

[16] Ghosh-Dastidar M., Dworakowski R., Lioupis C., Maccarthy P., Valenti D., El Gamel A., Monaghan M., Wendler O. (2011). The combined treatment of aortic stenosis and abdominal aortic aneurysm using transcatheter tecniques: A case report. J. Cardiovasc. Surg..

[17] Horiuchi Y., Izumo M., Kusuhara T., Yokozuka M., Taketani T., Tanabe K. (2017). Combined transcatheter aortic valve implantation and type II endoleak repair for abdominal aortic aneurysm. Cardiovasc. Interv. Ther..

[18] Kawashima H., Watanabe Y., Kozuma K. (2017). Successful transfemoral aortic valve implantation through aortic stent graft after endovascular repair of abdominal aortic aneurysm. Cardiovasc. Interv. Ther..

[19] Chakraborty B.R., Greason K.L., Oderich G.S., Bresnahan J.F., Reeder G.S., Suri R.M., Mathew V., Rihal C.S. (2013). Endovascular abdominal aortic aneurysm repair to facilitate access for transcatheter aortic valve implantation. Ann. Thorac. Surg..

[20] Aluko Y., Diehl L., Jacoby R., Chan B., Andrews S., McMillan E., Sharkey K., Shook P., Ntim W., Bolorunduro O. (2015). Simultaneous transcatheter aortic valve replacement and endovascular repair for critical aortic stenosis and large abdominal aortic aneurysm. Cardiovasc. Revasc. Med..

[21] Binder R.K., Maisano F., Lachat M. (2015). First report of simultaneous transcatheter aortic valve replacement, endovascular aortic aneurysm repair, and permanent pacemaker implantation after multi-vessel coronary stenting and left atrial appendage occlusion. Eur. Heart J..

[22] Marchi F., Cerillo A.G., Rizza A., Mariani M., De Caterina A.R., Palmieri C., Maffei S., Berti S. (2015). Large abdominal aortic aneurysm in a high-risk surgical patient: Combined percutaneous transfemoral TAVI and EVAR procedure. J. Heart Valve Dis..

[23] Sato Y., Horiuchi Y., Yahagi K., Okuno T., Kusuhara T., Yokozuka M., Miura S., Taketani T., Tanabe K. (2018). Simultaneous transcatheter aortic valve implantation and endovascular aneurysm repair in a patient with very severe aortic stenosis with abdominal aortic aneurysm. J. Cardiol. Cases.

[24] Gotzmann M., Hehen T., Germing A., Lindstaedt M., Yazar A., Laczkovics A., Mumme A., Mügge A., Bojara W. (2010). Short term effects of transcatheter valve implantation on neurohormonal activation, quality of life and 6 min walk test in severe and symptomatic aortic stenosis. Heart.

[25] Walker T.G., Kalva S.P., Yeddula K., Wicky S., Kundu S., Drescher P., d’Othee B.J., Rose S.C., Cardella J.F. (2010). Clinical practice guidelines for endovascular abdominal aortic aneurysm repair: Written by the Standards of Practice Committee for the Society of Interventional Radiology and endorsed by the Cardiovascular and Interventional Radiological Society of Europe and the Canadian Interventional Radiology Association. J. Vasc. Interv. Radiol..

[26] Nishimura R.A., Otto C.M., Bonow R.O., Carabello B.A., Erwin J.P., Fleisher L.A., Jneid H., Mack M.J., McLeod C.J., O’Gara P.T. (2014). 2014 AHA/ACC guideline for the management of patients with valvular heart disease: A report of the American College of Cardiology/American Heart Association Task Force on practice guidelines. Circulation.

[27] Oh S., Kim J.H., Jeong M.H. (2021). Minimally invasive transcatheter aortic valve replacement and sequential repair of abdominal aortic aneurysm in an octogenarian. Chonnam Med. J..

[28] Yammine H., Briggs C., Rolle Q., Ballast J., Frederick J., Skipper E., Downey W., Rinaldi M., Scherer M., Arko F. (2021). Reemplazo transcatéter de válvula aórtica y reparación endovascular de aneurisma aórtico simultáneos. J. Am. Coll. Cardiol..

[29] Nunes J., Tinoco E., Gallo E., Pereira M., Hipólito L., Silva A. (2019). Severe aortic stenosis associated to large abdominal aortic aneurysm: Concomitant treatment with transcatheter aortic valve replacement and endovascular aneurysm repair. J. Transcatheter Interv..

